# Screening Pregnant Women and Their Neonates for Illicit Drug Use: Consideration of the Integrated Technical, Medical, Ethical, Legal, and Social Issues

**DOI:** 10.3389/fphar.2018.00961

**Published:** 2018-08-28

**Authors:** Hayley R. Price, Abby C. Collier, Tricia E. Wright

**Affiliations:** ^1^Faculty of Pharmaceutical Sciences, The University of British Columbia, Vancouver, BC, Canada; ^2^Department of Obstetrics, Gynecology and Women’s Health, John A. Burns School of Medicine, University of Hawaii, Honolulu, HI, United States; ^3^Department of Psychiatry, John A. Burns School of Medicine, University of Hawaii, Honolulu, HI, United States

**Keywords:** addiction, neonate, neonatal abstinence syndrome, pharmacokinetics, relational ethics and care

## Abstract

North America is currently suffering from one of the worst epidemics of illicit drug use in recent history: the opioid crisis. Pregnant women are not immune to the ravages of substance misuse which affects themselves, their pregnancies, and the wider community. The prevalence of drug misuse in pregnancy is not well quantified due to the lack of good validated tests, cooperation between clinicians and scientists developing tests, and consensus as to who should be tested and how results should be used. A wide range of tissues can be tested for drug use, including maternal blood, urine, and hair; neonatal meconium, urine, and hair; and placenta and umbilical cord tissues. Testing methods range from simple spectrophotometry and clinical chemistry to sophisticated analytical HPLC or mass spectrometry techniques. The drive for ever greater accuracy and sensitivity must be balanced with the necessities of medical practice requiring minimally invasive sampling, rapid turnaround, and techniques that can be realistically utilized in a clinical laboratory. Better screening tests have great potential to improve neonatal and maternal medical outcomes by enhancing the speed and accuracy of diagnosis. They also have great promise for public health monitoring, policy development, and resource allocation. However, women can and have been arrested for positive drug screens with even preliminary results used to remove children from custody, before rigorous confirmatory testing is completed. Balancing the scientific, medical, public health, legal, and ethical aspects of screening tests for drugs in pregnancy is critical for helping to address this crisis at all levels.

## Introduction

In the 21st century, North America has experienced an increase in the use of prescription and non-prescription opioids, so large and rapid that it has become an epidemic (Okie, 2010; Metz et al., 2018). Millions of people are affected by this epidemic, which does not discriminate by age, gender, race, geographic area, or socio-economic status (Manchikanti et al., 2012; Metz et al., 2018). Within the wider population, pregnant women are not immune to substance use disorders and it is estimated over 10% of births in the United States each year are affected by illicit drug or alcohol use (Wabuyele et al., 2018). *In utero* exposure to drugs puts the fetus at risk of premature delivery, physical, and cognitive developmental problems, and can increase the risk of neonatal mortality (Chasnoff, 1988; Chasnoff et al., 1992; Stover and Davis, 2015). Additionally, exposure may cause neonatal abstinence syndrome (NAS), in which the fetus develops symptoms of withdrawal following delivery (Kocherlakota, 2014; Stover and Davis, 2015). Since 1999, the incidence of NAS is estimated to have increased by over 300%, coinciding with the onset of today’s opioid epidemic (Ko et al., 2016).

The prevalence of drug use in pregnancy is difficult to quantify. There are no specific guidelines for drug screening in pregnancy, and when combined with unreliable self-reporting, the true numbers of affected births are unknown. Approximately 5% of women will admit to taking illicit drugs while pregnant (Substance Abuse and Mental Health Services Administration, 2014). However, self-reported studies regarding maternal substance use disorders have a high incidence of underreporting, due to fear and associated stigma (Stone, 2015). Many women fear the consequences of using drugs in pregnancy, even if they cannot stop. Certain US States including Alabama, South Carolina, and Tennessee have charged women who have taken drugs in pregnancy with child abuse and mothers who use illicit drugs are at high risk of losing custody of their newborns (Young et al., 2007; Stone, 2015). While these laws were originally designed to protect the fetus, the fear instilled in women can prevent them from enrolling in treatment programs and accessing medical care throughout pregnancy. This has opened the debate regarding drug screening in pregnancy: who should be screened, by which methods, and how should these results be used?

Currently, there are few validated and standardized tests for drug screening in pregnant women (Grekin et al., 2010; Lam et al., 2015). This is combined with a general absence of cooperation between scientists, who develop and validate these tests, and clinicians who use and interpret them. This leads to considerable uncertainty as to when testing should occur, which tests should be used, and how testing should be implemented. A wide range of maternal and fetal tissues are available for screening, including maternal and fetal blood, urine and hair, and neonatal meconium, as well as reproductive tissues such as placenta and umbilical cord. Because discrepancies in the results from different tissues have been observed, this has added to the debate around testing. For example, Montgomery et al. (2006) found that the levels of opioids in umbilical cord corresponded well to the levels found in meconium. On the other hand, a recent study comparing the levels of five drug classes in a large number of matched umbilical cord and meconium samples found the results were highly discordant (Colby, 2017). The utility of each tissue as a screening matrix is dependent on a number of factors; different tissues will provide insight into drug use at varying times in pregnancy, a drugs’ physicochemical characteristics and pharmacokinetic profiles should be considered as this will affect the suitability of various tissues (e.g., blood vs. meconium), and the specificity and sensitivity of the analytical method being used also affects outcomes.

Screening methods can employ a number of different techniques spanning spectrophotometry, enzyme-linked immunosorbent assays (ELISAs) and sophisticated gas chromatography (GC) or high-performance liquid chromatography (HPLC) coupled to various detection techniques such as mass spectrometry (MS). The results of some techniques provide evidence of substance use, but further testing using more sensitive techniques is then required to confirm and quantify drug presence. In medical practice, there is a need for techniques that have a rapid turnaround time and are practical for use in a clinical laboratory. Speed and accuracy of test results are vital for early diagnosis of mothers and their neonates to aid in treatment decisions, yet this must also be balanced with sensitivity and quantitation. As an example, a mother ingesting poppy seeds (such as in poppy seed cake) may test positive for morphine and codeine from a screening test, and testing for more specific markers is required to differentiate between poppy seeds and illicit drug use such as heroin (Hayes et al., 1987; Lachenmeier et al., 2010).

Finally, there are several ethical, legal, and social considerations around screening for drug use in pregnancy that can be lost in the rush to test and confirm from a scientific and/or analytical perspective. The issue of consent is often raised in terms of drug screening; which in the specific case of pregnancy is complicated because the drug use affects both the mother and neonate. Additionally, the ethical principal of “respect for persons” mandates that the woman give consent for the procedure given the social and legal ramifications of the test (Zizzo et al., 2013). Women also fear stigmatization and the legal consequences of drug use in pregnancy. The legal issues pertaining to this are dependent on state and federal laws, which can also place clinicians in difficult positions related to screening. The official position of the American Society of Addiction Medicine (ASAM) and the American College of Obstetricians and Gynecologists is that all women should be screened using a validated screening test, and not biochemical measures (ACOG Committee on Health Care for Underserved Women and American Society of Addiction Medicine, 2012; American Society of Addiction Medicine, 2017). Decisions regarding the type of screens being performed, and the results of screening, need to be made based on a balance between scientific, medical, public health, legal, and ethical considerations around drug testing.

## Scientific Approaches to Screening

Over the last several decades, there have been great advances in the tools available for drug screening (Ombrone et al., 2016) and tests are now capable of giving more accurate and precise results than ever before. Traditionally, drug testing has been performed using ELISAs (Wabuyele et al., 2018). This method has advantages in that it is relatively cheap, easy to perform in a general laboratory, and has a rapid turnaround time. Additionally, because ELISA has been used for many years, there are multiple commercial antibodies available that are validated in many human tissues. The reliability of an ELISA depends on the tissue being tested and the drugs being detected in that tissue. For certain tests, including cocaine metabolites in urine, ELISA is a reliable method (Cone, 1989). However, for other drug and tissue combinations there is a relatively high incidence of both false positives and negatives (Yee and Wu, 2011). A positive ELISA in maternal tissue should be flagged for further screening, and presence of the drug confirmed by a more specific test.

Due to the limitations of immunoassays, biomedical and analytical scientists strive for more sensitive techniques capable of accurately quantifying drug concentrations. As such, the gold standard technique for drug screening is GC or liquid chromatography (LC) coupled to a mass spectrometer (MS). GC-MS, LC-MS, LC-MS/MS, and LC-time-of-flight-MS (LC-TOF-MS) techniques are extremely sensitive, and capable of detecting compounds in the nanogram or even picogram range (Horning et al., 1973; Himes et al., 2013). With limits of detection this low, drugs can be detected for longer periods of time following last dose, including well below therapeutic levels. Ideally, a positive immunoassay will be confirmed using one of these analytical techniques, thereby eliminating false-positives. However, mass spectrometers are very expensive, running to many hundreds of thousands of (US) dollars and few clinical laboratories have access to them. Additionally, the setup and validation of methods is very technical, and requires a trained spectrometry technician to design novel methods. While increased accuracy and quantitation is important, this needs to be balanced with rapid turnaround time and techniques that are available and can reasonably be carried out in a clinical setting. At this time, GC-MS and LC-MS techniques are primarily confined to specialized Academic and Forensic testing laboratories and are not practical for hospital or clinical laboratory service (CLIA) laboratories.

Regardless of the method being used for drug screening, there is also an urgent need for standardized tests that are validated for drug screening in pregnant women (Moeller et al., 2008; Substance Abuse and Mental Health Services Administration, 2017). The only standardized drug screening tools used in clinical practice that are also common during the hiring process of many companies, are immunoassay urine drug screens capable of detecting amphetamines, THC, cocaine, opiates, and phencyclidine as outlined by the Substance Abuse and Mental Health Services Administration (2017; Moeller et al., 2008). These assays detect only recent drug exposure and are not necessarily a good indicator of cumulative fetal drug exposure because the extent of placental drug transfer may be unknown. In addition, these five drugs are not always the ones women use. Correctly validated screening techniques for drug use in pregnancy should be the goal for use in clinical laboratories across the country, and worldwide. The FDA guidelines for validation of bioanalytical techniques state that validation includes evaluation of accuracy, precision, selectivity, sensitivity, reproducibility and stability (U.S. Department of Health and Human Services, 2013). A correct and thorough validation ensures that the technique is reliable, repeatable, and is accurately measuring what is intended. Moreover, each method needs to be re-validated for use in different tissues, and a partial-validation must be performed when setting up the method in a new laboratory (U.S. Department of Health and Human Services, 2013).

## Timing of Screening

As mentioned, universal screening for pregnant women has been recommended (ACOG Committee on Health Care for Underserved Women and American Society of Addiction Medicine, 2012; American Society of Addiction Medicine, 2017). In part, this stance arises from the opioid epidemic that spans age, race, and socioeconomic status. However, clinicians face real dilemmas surrounding this stance, not least is the decision of when screening should occur. Ideally, screening should be performed as early as possible in prenatal care so that clinicians are aware of their patient’s substance use and can make informed decisions for prenatal care as well as facilitating entrance into addiction treatment (if necessary and desired) thereby limiting the duration of exposure to the fetus. For example, physicians providing fertility treatments to women have the unique opportunity to screen women before they conceive, potentially preventing fetal exposure (Wright, 2017). During prenatal care, physicians have the opportunity to assess the risk of drug exposure by opening a discussion about past substance use habits or by using a questionnaire. There are a number of validated screening tools, including the Alcohol Use Disorders Identification Test (AUDIT-C), a three-question test for alcohol use (Bradley et al., 2003) and the 4 P’s test for substance use (Chasnoff et al., 2007). However, self-reported drug use is often unreliable, especially in a situation such as pregnancy where fear and stigma may result (Ostrea et al., 1992; Grekin et al., 2010), bringing into question the validity of these screening tools. Screening may be initiated during pregnancy based on risk factors including previous known drug use, previous birth complications, delays in accessing prenatal care, or frequently missing prenatal appointments. Using risk factors as a method for deciding who needs to be screened introduces the potential for bias as the physician decides who should be tested on a relatively arbitrary basis.

Another opportunity for drug screening to occur is after birth, using maternal and fetal samples, or associated reproductive tissues such as the placenta and umbilical cord which are normally discarded (Gareri et al., 2006). Post-birth screening may be a useful tool if the neonate has been admitted to the neonatal intensive care unit (NICU), with signs of NAS. In these cases, a drug screen provides the clinician with information about what substances the fetus may have been exposed to, guiding treatment options and leading to improved neonatal outcomes (Cotten, 2012). Situations such as this require an accurate test with a rapid turnaround time, to ensure an accurate diagnosis and treatment.

## Types of Screening for Illicit Drugs in Pregnant Women

There are a wide range of tissues available for screening drug use in pregnancy, each with their own advantages and disadvantages. Maternal tissues including blood, urine and hair can be screened before or after birth, and fetal tissues such as blood, urine, hair, and meconium can be screened following birth. Additionally, reproductive tissues such as the placenta and umbilical cord present potential screening matrices. Different matrices may provide insight into drug use at various times throughout pregnancy. So, in addition to choosing an acceptable tissue for screening, there are a number of different testing methods that can be used. Here, we will discuss screening methods by sample type, and highlight the advantages and disadvantages of each.

### Maternal or Fetal Blood

Blood is one of the most commonly collected tissues, but has limited use as a drug screening tool in pregnancy, and otherwise. It may not provide insight into maternal substance use if the woman has ceased drug use prior to medical appointments when blood will be drawn (Cotten, 2012). In contrast, fetal blood is usually only taken from the umbilical cord after birth, as venous collection is difficult and invasive requiring a trained professional and only generally used in the case of a very select set of [suspected] fetal abnormalities (Westgate et al., 1994). However, both maternal and cord blood have a very short window of detection, and are rarely used clinically or in research for drug detection (Wright, 2015).

There are few studies that compare plasma drug levels to self-reported drug use, to investigate drug use in pregnancy. One Swedish study performed by Wolgast et al. (2018) showed good concordance between reported drug use and drugs detected by LC-TOF-MS, in contrast to most studies which find a high incidence of under-reported drug use. In the Wolgast study, drugs that are only occasionally used, such as anti-histamines and analgesics; were detected at the highest frequency. However, these were also the most often misreported/under-reported drugs, likely due to their occasional use. Another recent study also used LC-TOF-MS to screen maternal plasma samples, where samples were anonymized and women were unaware of the screening (Aagaard et al., 2018). This research included multiple xenobiotics including illegal drugs, prescription drugs, indicators of smoking, and caffeine. According to the authors, ∼83% of women screened positive for xenobiotics. As an addendum to this study, blood levels of cotinine have been measured as a marker of maternal tobacco use multiply over the last 40 years (Ivorra et al., 2014); with high levels of cotinine in newborns of smoking mothers. Smoking and nicotine are outside of the scope of this particular paper, but it should be noted that monitoring for tobacco products is far advanced from other drugs and even more so than the illicit drugs we are referring to, due to decades of monitoring and outcomes research (Ivorra et al., 2014).

### Maternal Urine

Urine is the most commonly used matrix for drug screening in adults, including pregnant women, and is relatively easy and non-invasive to collect (Colmorgen et al., 1992). However, it has a short window of detection, providing insight only into recent exposure and may not be useful for detection of substance use disorders. It has been suggested that urine screening may be valuable early in pregnancy, when a woman visits a physician for prenatal care (Colmorgen et al., 1992). Early detection of maternal drug use presents the opportunity for intervention, thereby limiting exposure to the fetus. Unfortunately, urine drug screening has been shown to have a high incidence of false positives (Center for Substance Abuse Treatment, 2006), which may have serious consequences when screening for drug use in pregnancy. Urine samples are also easily adulterated, and women may wait to attend appointments until enough time has passed that the drug can no longer be detected in urine (Fu, 2016).

A number of validated methods are currently available for drug screens in urine. Immunoassay-based urine drug screens are available for common drugs of abuse including alcohol, amphetamines, benzodiazepines, marijuana, and cocaine (Moeller et al., 2008). However, there are a number of recognized agents which can cause false positive results (Moeller et al., 2008), and a MS method is recommended to confirm presence of a drug after a positive result from immunoassay. A screening study by Hoeke et al. (2015) compared the results of questionnaires to an untargeted LC-MS/MS urine screen. The authors observed very low agreement between self-reported results and urine screens, between 19 and 25% (Hoeke et al., 2015). In another study, urine samples were collected from neonates suspected to have been exposed to illicit drugs (Hon et al., 2016). Screening was performed using LC-TOF-MS, with a screening library of more than 300 drugs and metabolites. Using this method, drugs of abuse and other medications were detected in approximately 66% of neonatal urine samples (Hon et al., 2016). Perhaps one of current best-practice efforts is illustrated by Women’s and Children’s Hospitals in Cincinnati that have a universal screening program, and currently perform preliminary screening by immunoassay, followed by confirmation by MS (Newman, 2016). Their MS method is capable of screening for 47 drugs of abuse in a 6-min protocol, providing the opportunity for rapid turnaround time. This program was implemented because the Cincinnati region had experienced a sixfold increase of *in utero* drug exposure between 2009 and 2014 (Wexelblatt et al., 2015). The hospital had previously used a risk-based screening method, but 20% of the opioid positive results would have been missed if they had not implemented universal screening (Wexelblatt et al., 2015). The authors of this review point to the Cincinnati model as “Gold Standard” and one to be both admired and emulated in Hospitals around the world, as resources allow.

Urine sampling is therefore very useful in situations where a rapid diagnosis is needed, such as in a neonate suspected of having NAS, with the drawback that only recent maternal drug use can be detected. Regardless, because it is readily available and easily collected, it will likely remain the most important matrix used in drug screening.

### Meconium

Meconium is the first bowel movement of the fetus, typically passed in the first few days of life, which contains a number of metabolic waste products (Gareri et al., 2006). Meconium usually begins to form at the beginning of the second trimester as the fetus’ swallowing reflex begins, permitting the swallowing of amniotic fluid (Ostrea et al., 1989; Kwong and Ryan, 1997). Meconium is often considered the gold standard for drug testing in newborns, although (by definition) it cannot detect first trimester drug exposure. Meconium can be collected in a non-invasive manner, and also provides a longer window of detection because it begins to form around 12 weeks’ gestation and there may be an element of drug concentration in the meconium tissue (Ostrea et al., 1989). However, collection may be missed as meconium can sometimes be passed *in utero* and screening may show drugs administered during labor, potentially confounding results (Farst et al., 2011; Wood et al., 2014). Another drawback of meconium as a screening matrix is that it is not immediately available; if screening is intended to aid in diagnosing or treating a newborn in distress, meconium may not be an appropriate choice due to the length of time for the first bowel movement (Lozano et al., 2007). Additionally, drug exposure and low birth weight, often a consequence of *in utero* drug exposure, can delay the passage of meconium (Verma and Dhanireddy, 1993).

In terms of sensitivity, meconium has previously been considered the best tissue for evaluating fetal drug exposure. As such, there are a large number of methods available for screening across most drug classes, including cocaine, opioids, marijuana, methamphetamine, cotinine, and alcohol use (Wright, 2015). An LC-MS/MS method which detects amphetamines, opioids, opioid partial agonists, and metabolites of these drugs was developed by Ristimaa et al. (2010) along with another, separate, method for cannabinoid screening. Limits of detection ranged from 0.2 to 20.0 ng of drug per gram of meconium. Additionally, these same authors applied LC-TOF-MS to a drug library and 77 compounds were detected in meconium samples (Ristimaa et al., 2010). Another LC-MS/MS method has been published capable of screening 22 antidepressant and anxiolytic drugs (Pichini et al., 2016), as well as a method for screening 20 antiretroviral drugs and metabolites in a single analytical run (Himes et al., 2013). The majority of available methods for meconium screening are LC-MS based, and many others exist with shorter run times, screening drugs and metabolites specific to certain drug classes such as marijuana, nicotine, or alcohol use (Tynon et al., 2015; Prego-Meleiro et al., 2017).

Hence, meconium is most useful for detecting long-term maternal drug use (including dependence and misuse disorders) due to the long window of detection. Meconium is also valuable epidemiologically, for determining the true incidence of drug use in the population (as opposed to self-report that is confounded by recall bias, stigma, and untruthfulness) because meconium is highly specific and sensitive, and can be used as an analytical tool.

### Maternal or Neonatal Hair

Hair, both maternal and neonatal, is another useful tissue for drug screening. It has advantages as a screening tissue because it is easy to collect and has a long window of detection… up to months depending on the length of the hair (Lendoiro et al., 2013). Neonatal hair begins to protrude from the scalp around the beginning of the third trimester, and therefore may provide insight into third trimester drug use (Gareri and Koren, 2010; Lendoiro et al., 2013). Some drawbacks of hair as a screening tissue include: differential drug deposit in hair depending on hair type (Henderson et al., 1998), environmental contamination (Wright, 2015), limited amount of neonatal hair (Delano and Koren, 2012) or social or cultural objection to removal of hair (Lendoiro et al., 2013) and bald babies. Extensive sample extraction and manipulation is also required and certain drugs may extract poorly from hair (Lendoiro et al., 2013).

Despite these drawbacks, hair is one of the longest standing matrices for toxicology screening, and a number of methods are available. An evaluation of two different immunoassays for detecting cannabinoids, opiates, cocaine, amphetamines, benzodiazepines, and methadone in hair has been performed by Musshoff et al. (2012). Sensitivity of one immunoassay ranged from 91 to 98%, and specificity ranged from 72 to 89% across all drug classes, but the test was not useful for cannabinoid screening. The other immunoassay investigated was only useful for morphine and cocaine despite manufacturer recommendations. Subsequently, Lachenmeier et al. (2006) proposed a two-step method for opiates and cocaine screening in hair; initial screening by immunoassay followed up by GC-MS to confirm. Both methods were validated and the ELISA provided semi-quantitative results, while the GC-MS method was used to confirm presence of the drug and provide absolute quantitation. More recently, MS methods for screening multiple drug classes in hair have become available. Hegstad et al. (2008) developed an LC-MS/MS method for nicotine, opioids, antidepressants, and opioid maintenance therapy drugs. In 2012, a fully validated LC-MS/MS method was published for 35 analytes, including cannabinoids, opioids, amphetamines, cocaine, benzodiazepines, and other illicit drugs (Lendoiro et al., 2012). Limits of detection ranged from 0.2 to 50 pg/mg of hair and amounts as low as 50 mg of hair were required for analysis. While screening for marijuana using immunoassays did not yield good results, a GC-MS method for THC, cannabidiol, and cannabinol in hair was developed by Nadulski and Pragst (2007) with good sensitivity. Limits of detection were 0.012, 0.013, and 0.016 ng/mg of hair for THC, cannabidiol, and cannabinol, respectively. However, results showed that higher concentrations were found further out from the scalp, indicating the compounds can be incorporated into the hair outside of the hair shaft, an important issue to be considered in screening.

Collectively, hair as a matrix has utility when trying to determine long-term drug use. Hair is also a valuable matrix when a sample is required immediately, to aid in diagnosis of the neonate, and when it would not be practical to wait for meconium.

### Placenta

Reproductive tissues, such as the placenta, are other matrices available for screening. Placental tissue collection is non-invasive and simple because it is usually discarded after birth (Birdsong, 1998; Burns, 2014). The average placenta weights approximately 500–600 g, providing a large amount of sample for testing (Haavaldsen et al., 2012). This also means there is extensive tissue processing and sample cleanup required in order to have a sample ready for screening. A standardized procedure for sample preparation has not yet been developed and this impacts the differential sensitivities and accuracies across multiple laboratories world-wide. Although the placenta begins to form at approximately 4 weeks gestation, the window of detection has not been well defined (Lozano et al., 2007;Joya et al., 2010), for several drugs the detection window is believed to be relatively short – similar to blood (Cotten, 2012). However, for certain drugs and chemicals such as methadone (Malek et al., 2009; de Castro et al., 2011) or sufentanil (Johnson et al., 1997) the placenta has been demonstrated to act as a “sink,” bioaccumulating these xenobiotics across gestation.

The villous placenta has not been thoroughly investigated as a screening tissue, in part because of the extensive tissue processing required and lack of understanding of drug metabolism, distribution, and transport within and across the organ. However, there are some reports investigating the placenta as a screening tool in mothers undergoing opioid maintenance therapy using both buprenorphine and methadone (Concheiro et al., 2010; de Castro et al., 2011). In mothers given buprenorphine during pregnancy, levels of the drug and its metabolites were measured in placenta using LC-MS (Concheiro et al., 2010). The median concentration of buprenorphine was 1.6 ng/g, and these concentrations were 15- to 70-fold lower than in meconium and were undetectable in one placenta. In another study, methadone and the metabolite 2-ethylidene-1,5-dimethyl-3,3-diphenylpyrrolidine were measured by a validated liquid chromatography-ion trap MS method and positive correlations were observed between methadone levels in the placenta and mean daily dose (de Castro et al., 2011). In both studies, the placental tissue was also screened using a validated LC/MS method for cocaine, opiates and metabolites (de Castro et al., 2009). The authors later developed and validated an LC-MS/MS method for quantification of opiates, methadone, amphetamines, cocaine, and metabolites in placental tissue (de Castro et al., 2013). Limits of quantitation ranged from 1 to 5 ng/g and the method was applied to placentas from drug dependent pregnant patients. However, the precise dosage of drugs the mothers had taken were unknown, and many tissues screened positive but the amounts were below the limits of quantitation for the method.

### Umbilical Cord

The umbilical cord is a part of the placenta that connects the developing fetus to the placenta, and presents another potential tissue for screening. Like the villous placenta, it is easily accessible and non-invasive, and is available immediately after birth. It may represent a better choice than villous tissue as it exists completely on the fetal side of the vascular organ, so it may better reflect fetal exposure (Wright, 2015). However, drug levels in the umbilical cord have been shown to be lower than in matched meconium (Montgomery et al., 2006; Colby, 2017). It is uncertain whether this represents accurate results to maternal ingestion, or whether meconium might represent a cumulative exposure measure. The flow of drugs across the placenta, distribution of drugs in the umbilical cord, and the detection window for screening in this tissue are not completely understood.

There are a number of previously published methods for drug screening in umbilical cords. The agreement of umbilical cord cotinine levels and maternal self-report of smoking in pregnancy was investigated using ELISA to determine cotinine levels (Wright et al., 2011). The agreement between self-reported smoking and cotinine levels was fair; sensitivity was 27% and specificity was 98%. In this study, illicit drugs were screened using ELISA also, and the test was more sensitive for detecting marijuana than other drugs of abuse such as methamphetamine (Wright et al., 2011; Collier et al., 2015). Montgomery et al. (2006) assessed the agreement between meconium and umbilical cord screening for amphetamines, opioids, cannabinoids, and cocaine. Drug class-specific immunoassays were used to screen for each individual drug class, and positive ELISAs were confirmed for amphetamines using GC-MS assays. Agreement of cord and meconium samples were 96.6, 94.9, 99.2, and 90.7% for amphetamines, opiates, cocaine, and cannabinoids, respectively (Montgomery et al., 2006). The authors concluded that umbilical cords perform as well as meconium, and has advantages over meconium because of the rapid turnaround time and potential for epidemiological testing. However, in 2017 another study found cord tissue and meconium samples were highly discrepant (Colby, 2017). Six drug classes (amphetamines, cocaine, opioids, barbiturates, benzodiazepines, and cannabinoids) were investigated. Whilst agreement ranged from 76 to 100%, Cohen’s kappa coefficient, which accounts for agreement occurring by chance, was less than 65% for five of the drug classes investigated. On the other hand, Jones et al. (2015) published a novel and validated LC-MS/MS method for codeine, morphine, 6 monoacetylmorphine, and meconin in 2015. Umbilical cord was found to be a suitable tissue for the screening of these drugs, and limits of detection were greatly improved compared to previously available methods. Recently, a commercial ELISA for detection of opioids was validated in umbilical cord tissues (Knight et al., 2018). Absolute quantitation could not be determined due to cross reactivity in excess of 100% for certain opioid metabolites, but the method showed perfect selectivity for cords in the study that were deemed opioid positive by clinical diagnostics. Most recently, the umbilical cord has been shown to be a sensitive and specific screening tissue for cannabis use (Kim et al., 2018). The authors showed excellent concordance with meconium, and were able to absolutely quantitate tetrahydrocannabinol (THC) as well as several of its hydroxyl and glucuronide metabolites.

The umbilical cord has advantages as a screening tissue because it is easily accessible and readily available. It is considered clinical waste and is therefore universally available. Although its utility in screening some drug classes remains to be evaluated, the tissue has been used for a wide range of hydrophilic (methamphetamine, cotinine, and cocaine) as well as lipophilic (opioids and cannabinoids) drugs, it would appear that cords have wide applicability.

## Ethical, Legal, and Social Considerations in Screening

While drug use in pregnancy remains a critical public health issue, many legal, social, and ethical considerations are important for deciding when and how to screen pregnant patients. As we have discussed, risk-based screening is not recommended because of the potential for bias. The ethical principal of justice may require that universal screening be done (Zizzo et al., 2013). However, in public health, screening is usually ethically differentiated as “with” or “without” consent (Moreno and Minkoff, 1992). It is generally believed that screening with consent does not pose significant individual or public health risks, as long as correct and informative follow-up is undertaken. As mentioned, respect for persons requires that informed consent be obtained when screening for drug use, given the multitude of legal and social implications of a positive screen. Consuming drugs in pregnancy is considered child abuse in at least 19 states in the United States, and women can lose custody of their children based on a positive screening test, even without confirmation (Stone, 2015). Because women know of these legal consequences, many will engage in behavior to avoid detection, including not presenting for prenatal care and attempting to deliver outside of the hospital environment (Stone, 2015). Therefore, screening women without adequate protection for their legal and social rights can have negative effects on both maternal health and the health of their children. Examples of this in a previous context come from the HIV epidemic, where pregnant women routinely chose not to be screened due to the stigmatizing nature of the diagnosis. Subsequent programs were often presented as “universal screening” to de-stigmatize this process. However, upon diagnosis women commonly became resentful and refused to co-operate with their clinicians, thereby having negative healthcare outcomes attributable to testing itself (Moreno and Minkoff, 1992). Another directly relatable consequence of screening that can be taken from the HIV epidemic is that earlier in the epidemic, HIV diagnosis carried no cure and/or inadequate therapy. This is paralleled in the case of addiction because there is no cure for addiction, so screening may be considered as a stressor because it can identify, but not resolve the primary medical issue and requires ongoing attention.

An additional concept is respect for autonomy – a guiding principle in clinical ethics. Respect for autonomy means that the clinician must create an environment in which the patient is empowered to make informed decisions (Mark and Terplan, 2017). This is not the same as beneficence where the clinician performs actions to the benefit of the patient. In the context of drug use in pregnancy, most literature surrounding respect for autonomy relates to women with chronic diseases such as schizophrenia (Dudzinski and Sullivan, 2004) or epilepsy (Beran, 2006) that subsequently become pregnant. One relevant and very recent paper has examined pregnancy in the context of increasing cannabis decriminalization/legalization and respect for autonomy in these women (Mark and Terplan, 2017). The authors report that although most women cut back or stop cannabis use in pregnancy, health care providers are relatively poor at counseling women and providing accurate clinical and scientific information to these patients. In part this is due to a lack of good information available from both scientific and clinical realms – an issue we have alluded to above, but the authors also identify a need to avoid a punitive atmosphere, including that risks to the fetus and mother should neither be minimized, nor overstated.

Increasingly, clinicians are finding themselves in need of tools relating to drug-use in pregnancy that can cope with these women as a special population. Many of the newer approaches to treating drug misuse such as safe injection sites have been shown to save lives in the general drug using population (Kaplan, 2018), but there has been little attention as to how this may be effective for pregnant women. One promising area, in our experience; has been implementation of harm-reduction models for substance misuse in pregnant women (Wright et al., 2012). Tricia Wright has been running the Perinatal Addiction Treatment Clinic of Hawaii (PATH) since 2007 based on a harm-reduction model, integrating addiction medicine, child-care, family planning, perinatal care, motivational incentives, social services, and transportation. These women show better pregnancy outcomes, improved self-care, lower rates of drug misuse and higher rates of parenting their children (Wright et al., 2012). Because drug misuse does not exist in isolation, treatment that encompasses ancillary contributors such as poverty, inter-personal violence, poor access to healthcare in general and psychiatric care specifically, and building of positive life-skills, without focusing on or mandating abstinence can be very effective in this vulnerable group.

A positive drug screen affects not only the woman and her child or children, but can extend to spouses, parents, and other family members (Wright, 2018). Often, both the woman and her partner misuse substances together. In other cases, a woman may hide her substance misuse from family and friends (Stone, 2015). If the substance misuse comes to light during a pregnancy drug screen, this can negatively impact a woman’s relationships with family and friends that by extension, are also her support networks to contend with the psychological and physical symptoms associated with dependence. Moreover, in cases where legal action is taken and/or children are removed from the mother’s custody, this leaves a very difficult, and emotionally and legally fraught decision as to where the children should ultimately be sent for care. Children can and have been sent to relatives, have entered temporary custody, foster care or even temporarily housed in juvenile detention facilities (Wright, 2018). These policies can do lasting damage to children and families. Michael Wald said it eloquently, “Removing a child from his family may cause serious psychological damage – damage more serious than the harm intervention was supposed to prevent” (Wald, 1975).

In this sphere, the field of relational ethics – a qualitative research method – appears to be making inroads. Relational ethics, broadly defined; seats ethical decisions and considerations in the context of a relationship. For the purposes of this article, that relationship is the clinician/patient relationship in the context of pregnancy and substance misuse. Soderstrom and Skolbekken (2012) studied women who had been incarcerated under the Norwegian penal code for using drugs in pregnancy, and how the incarceration affected them and their birth outcomes. The authors found profound effects on women’s preparedness for motherhood, and also that interventions required much higher clinician awareness of ethical challenges. The authors recommended that care of these women should be seen as a “therapeutic alliance” rather than an act of coercion or beneficence. Indeed, recently Campbell (2018) has argued that the standpoint should be of shared responsibility for healthy births. Her in-depth discussion sheds light on the ways in which medicalization and criminalization of substance abuse has increased surveillance and scrutiny of women. The prevailing argument of child protection, often fails to also consider reproductive and natural justice when incarceration is the preferred option. In this manner, the concepts of discrimination and stigmatization should be examined when illicit substance use is discovered through screening. It is known that drug use and misuse crosses all educational, socio-economic and geographic boundaries (Manchikanti et al., 2012; Metz et al., 2018). However, those from lower socio-economic or educational backgrounds are often more stigmatized by diagnosis. This effect seems to be prevalent in both universal health care systems such as Canada (Finnegan, 2013), and user-pays health systems such as the United States (Tauger, 2018). In Canada this has meant that certain groups become discriminated against, most notably indigenous peoples, whilst in the United States fractures appear most commonly along socio-economic lines. Screening should not create, in and of itself; marginalized groups.

Significant stress, both mental and financial, also occurs when legal actions are taken. Court fees and loss of income, whether by taking time off work directly for the woman concerned and/or her family for lawyer and court appointments, as well as the costs of being incarcerated are considerable economic stresses (Flavin and Paltrow, 2010). Meanwhile, the costs of housing, childcare, and other basic needs still need to be met. Unfortunately, due to the stigma and guilt of substance use in pregnancy, these issues will continue to be faced until more broadly based community and family support solutions are found. Viewing substance use disorders as medical issues that need to be addressed clinically, instead of criminal acts, would decrease stigma and lead to better treatment options for women (Flavin and Paltrow, 2010). Without the fear of being punished and/or the associated feelings of guilt, it is more likely that these women will access appropriate pre-natal care as well as treatment services and counseling which can improve outcomes for mothers and their children.

## General Discussion and Conclusion

Until recently the general focus on drug risks in pregnancy has been solely toward the fetus, e.g., malformations, developmental delay or, complications of pregnancy such as pre-term birth and intra-uterine growth restriction. Partly because of this, we lack good understanding of the pharmacokinetics of drugs in pregnancy and the role of the maternal-placental-fetal unit(s), their ability to clear and eliminate substances and/or preferentially concentrate them toward the developing child, and the subsequent consequences of these dynamics.

Despite a clear need for tests, as evidenced by the current opioid epidemic; our lack of pharmacological knowledge has been compounded by a general misunderstanding of addiction and substance use/misuse within the medical profession that is further complicated with respect to pregnant women and children. Misunderstanding is based on a lack of addiction knowledge in primary healthcare providers as well as a lack of evidence-based knowledge of drugs in pregnancy and the neonate. Moreover, local, state and federal policies tend to focus on the (generally unproven) risks of illicit drugs, while ignoring the real need for medication and medical care for pregnant women, such as for medical pain at the end of pregnancy due to physiological stress (Price and Collier, 2017). And then, in a punitive legal atmosphere; drug use and misuse cannot be treated as a medical issue and becomes increasingly politicized, legalized and stigmatized in these pregnant women and for their children.

We hope to have stimulated further dialog around (1) the accuracy, precision and applicability of testing modalities, (2) the desirability and need, for population screening from an individual and epidemiological point of view, and (3) critical ethical and social issues that inform the need for, and response to, testing pregnant women for illicit drug use and misuse. Universal testing, although advocated by some, is almost certainly problematic from societal standpoint and, as in the case of relational ethics (discussed above) new more patient-centered approaches to screening, diagnosis and medical care are necessary.

## Author Contributions

All the authors provided significant intellectual and technical input into the writing formatting and editing of the manuscript.

## Conflict of Interest Statement

The authors declare that the research was conducted in the absence of any commercial or financial relationships that could be construed as a potential conflict of interest.
